# Immunotherapy plus induction chemotherapy followed by radiotherapy alone or one cycle of concurrent chemotherapy with cisplatin vs induction chemotherapy followed by two cycles of concurrent chemotherapy with cisplatin in locoregionally advanced nasopharyngeal carcinoma

**DOI:** 10.3389/fimmu.2026.1766797

**Published:** 2026-05-25

**Authors:** Renba Liang, Xinxiao Li, Manyi Zhu, Fengming Lan, Fangmeng Fu, Ling Lei, Teng Zou, Li Ma, Peng Chen, Lingzhi Luo, Jing Jin, Jianghu Zhang

**Affiliations:** 1 Department of Radiation Oncology, National Cancer Center/National Clinical Research Center for Cancer/Cancer Hospital & Shenzhen Hospital, Chinese Academy of Medical Sciences and Peking Union Medical College, Shenzhen, China; 2 Department of Radiation Oncology, Shenzhen Luohu People’s Hospital, Shenzhen, China

**Keywords:** cisplatin, concurrent chemoradiotherapy, immunotherapy, induction therapy, nasopharyngeal carcinoma

## Abstract

**Objective:**

To compare the efficacy and safety of immunotherapy combined with induction chemotherapy followed by radiotherapy alone or one cycle of concurrent chemoradiotherapy with cisplatin versus induction chemotherapy followed by two cycles of concurrent chemoradiotherapy with cisplatin in patients with locally advanced nasopharyngeal carcinoma.

**Methods:**

A total of 173 patients with locally advanced nasopharyngeal carcinoma treated at our hospital from November 2019 to September 2024 were retrospectively analyzed. Patients received induction therapy with immunotherapy (toripalimab or tislelizumab) combined with chemotherapy (TPC or GP) followed by radiotherapy alone or one cycle of concurrent chemoradiotherapy with cisplatin (immunochemotherapy group). Patients received TPC or GP induction chemotherapy followed by 2 cycles of concurrent chemoradiotherapy with cisplatin (IC-CCRT group). Immunotherapy was given on day 1 of each cycle of induction therapy. The efficacy and toxicities of the two groups were evaluated.

**Results:**

After induction therapy, 20 patients (29.4%) in the immunochemotherapy group and 15 patients (14.3%) in the IC-CCRT group achieved complete remission (p=0.02). At a median follow-up of 27.5 months, recurrence or metastasis occurred in 7.4%(5/68) of the patients in the immunochemotherapy group and 19.0%(20/105) of those in the IC-CCRT group. The 2-year event-free survival (EFS), overall survival (OS), locoregional recurrence-free survival (LRRFS) and distant metastasis-free survival (DMFS) in the immunochemotherapy group were 93.5% vs 81.1% (p=0.03), 100.0% vs 97.9% (p=0.3), 96.9% vs 91.4% (p=0.2), 95.0% vs 88.2% (p=0.1) compared with the IC-CCRT group. There were 28 cases (41.1%) in the immunochemotherapy group and 58 cases (55.2%) in the IC-CCRT group experienced grade 3–4 acute adverse events. In the immunochemotherapy group, 4 patients developed grade 3–4 immune-related adverse events.

**Conclusions:**

Compared with induction chemotherapy followed by two cycles of concurrent chemoradiotherapy with cisplatin, immunotherapy combined with induction chemotherapy followed by radiotherapy alone or one cycle of concurrent chemoradiotherapy with cisplatin may improve the EFS of patients with locally advanced nasopharyngeal carcinoma with low adverse events. However, this data requires prospective randomized controlled studies to be confirmed in the future.

## Introduction

Nasopharyngeal carcinoma is a malignant tumor that occurs in the mucosa of the nasopharynx. More than 70% of the patients presented with locally advanced disease at the time of diagnosis and required concurrent chemoradiotherapy based on cisplatin (1). However, high-dose (2–3 cycles) concurrent cisplatin chemotherapy has significant toxic side effects, such as nausea and vomiting, mucositis, hematological cytotoxicity and causes irreversible injury to hearing, peripheral nerves, and kidney function. Moreover, cisplatin-chemotherapy can increase hospitalizations and treatment costs; rise the need for chemotherapy-related supportive measures, such as high-volume hydration (2, 3). Additional PD-1 blockade can increase the event-free survival of patients with locoregionally advanced NPC (4–6). Furthermore, a recent randomized study has shown that after induction chemotherapy for locoregionally advanced nasopharyngeal carcinoma, radiotherapy alone was noninferior to chemoradiotherapy in terms of 3-year progression-free survival with lower grade 3 to 4 short-term toxic effects (7). Interestingly, studies found that immunotherapy combination therapy without concurrent cisplatin was a feasible strategy with high therapeutic effect in failure-free survival and low toxicity in locoregionally advanced NPC (3, 8). However, application of immunotherapy through the whole course will increase adverse reactions. Therefore, this study assessed the efficacy and safety of omitting concurrent cisplatin or reducing the dosage of concurrent cisplatin after combination of induction chemotherapy and immunotherapy in locoregionally advanced NPC.

## Materials and methods

### Patients

This study retrospectively analyzed the data of patients with locally advanced NPC (stage III/IVa AJCC 8^th^ excluding T3-4N0M0) treated at the National Cancer Center/National Clinical Research Center for Cancer/Cancer Hospital & Shenzhen Hospital between November 2019 and September 2024. The inclusion criteria were as follows: (i) age ≥18 years; (ii) pathologically diagnosed with NPC; (iii) stage III/IVa in accordance with the 8th Edition of the AJCC; (iv) Karnofsky performance score (KPS) ≥70; (v) receiving induction chemotherapy (IC) or IC plus toripalimab (or tislelizumab) followed by RT or CCRT; (vi) receiving concurrent chemotherapy with cisplatin; (vii) receiving IMRT and (viii) IC including GP or TPC regimen. The exclusion criteria were as follows: (i) received concurrent immunotherapy; (ii) received GP or TPC adjuvant chemotherapy or adjuvant immunotherapy after RT or CCRT; (iii) underwent surgery before IC; (iv) T3-4N0M0; or (v) had a second malignancy.

### Treatment

The IC regimens were gemcitabine (1000 mg/m^2^ on days 1 and 8) and cisplatin (80 mg/m^2^ on day 1) (called GP) or nab-paclitaxel (200 mg/m^2^ on days 1), cisplatin (60 mg/m^2^ on day 1) and capecitabline (1000 mg/m^2^ twice every day on day 1-14) (called TPC) every 3 weeks for 2–3 cycles. Concurrent chemotherapy regimen was cisplatin (100 mg/m^2^ on day 1) every 3 weeks for 0–2 cycles. Toripalimab (240 mg) or tislelizumab (200 mg) was given intravenously on the first day of each cycle of induction chemotherapy. If patients received adjuvant capecitabine, capecitabine was given as followed: 1000 mg/m^2^ twice daily for 14 days every 3 weeks for 8 cycles.

After induction therapy, patients underwent nasopharyngeal and cervical magnetic resonance imaging and nasopharyngeal endoscopy to evaluate the efficacy. Then patients received radical radiotherapy as previously described (9). In brief, the radiotherapy protocol was as follows: 69.96 Gy at 2.12 Gy/fraction to the planning target volume (PTV) of the nasopharyngeal gross tumor volume and metastatic lymph nodes, 60.06 Gy to the PTV of the high-risk clinical target volume, and 54.45 Gy to the PTV of the low-risk clinical target volume. If patients received concurrent targeted therapy, nimotuzumab (200 mg) was used intravenously on the first day of radiotherapy every week.

### Clinical endpoints

The primary endpoints included event-free survival (EFS, the time from the start of treatment to disease progression or death from any cause). The secondary endpoints included overall survival (OS, the time from the start of treatment to death from any cause), distant metastasis-free survival (DMFS, the time from the start of treatment to distant metastasis) and locoregional recurrence-free survival (LRFS, the time from the start of treatment to locoregional recurrence) and toxicities. Efficacy evaluation was performed according to the Response Evaluation Criteria in Solid Tumors version 1.1 (RECISTv1.1). Acute toxicities were evaluated based on the Common Terminology Criteria for Adverse Events (version 4.0), and late toxicities of radiotherapy were evaluated on the basis of the Late Radiation Morbidity Scoring Scheme of the Radiation Therapy Oncology Group.

### Statistical analysis

Categorical variables were compared using chi-squared test or Fisher’s Freeman–Halton test. The numerical variables were evaluated by the one-way analysis of variance (ANOVA). The survival data were analyzed through Kaplan–Meier curves and log-rank tests. A multivariate Cox proportional hazards model was used to calculate hazard ratios (HRs), 95% confidence intervals (CIs) and independent prognostic factors. p<0.05 was considered statistically significant.

## Results

### Patient characteristics

A total of 173 patients were included in this study, including 68 cases who received radiotherapy alone or one cycle of concurrent chemoradiotherapy with cisplatin after induction chemotherapy and immunotherapy, and 105 cases who underwent 2 cycles of concurrent chemotherapy after induction chemotherapy. The characteristics of the patients are summarized in **Table 1**. Most patients were at N2 or N3 stage of the lymph nodes or advanced stage of the nasopharyngeal lesion. More than 60% of the patients were in stage IVA in two groups according to the 8th edition of the AJCC. There was no statistically significant difference in age, sex, T stage, N stage, TNM stage, pathological characteristics, cycles of induction therapy, adjuvant therapy, or concurrent nimotuzumab between two groups.

**Table 1:** Basic characteristics of patients.

Characteristics	Im+IC (N=68) (N, %)	IC (N=105) (N, %)	P-value
Gender			
Male	53(77.9)	77(73.3)	0.59
Female	15(22.1)	28(26.7)	
Age, years (Median, Range)	47(21-70)	44(19-69)	
Histology (nonkeratinizing)			
differentiated	5(7.4)	1(1.0)	0.11
undifferentiated	63(92.6)	104(99.0)	
T stage			
T1	10(14.7)	15(14.3)	0.77
T2	6(8.8)	14(13.3)	
T3	32(47.1)	43(41.0)	
T4	20(29.4)	33(31.4)	
N stage			
N1	12(17.6)	13(12.4)	0.33
N2	25(36.8)	50(47.6)	
N3	31(45.6)	42(40.0)	
Overall stage			
III	25(36.8)	41(39.0)	0.24
IVA	43(63.2)	64(61.0)	
IC cycle			
Two	42(61.8)	65(61.9)	1.00
Three	26(38.2)	40(38.1)	
IC regimen			
GP	54(79.4)	74(70.5)	0.22
TPC	14(20.6)	31(29.5)	
Im regimen			
Toripalimab	31(45.6)	N/A	
Tislelizumab	37(54.4)	N/A	
Concurrent chemotherapy cycle			
None	26(38.2)	0(0.0)	<0.0001
One	42(61.8)	0(0.0)	
Two	0(0.0)	105(100.0)	
Concurrent targeted therapy			
Yes	56(82.4)	80(76.2)	0.45
No	12(17.6)	25(23.8)	
Capecitabine adjuvant therapy			
Yes	5(7.4)	8(7.6)	1.00
No	63(92.6)	97(92.4)	

NA, not applicable. Im+IC, immunochemotherapy. IC, nduction chemotherapy. Im, immunotherapy.

### Efficacy

After induction treatment, 20 (29.4%), 43 (64.7%) and 4 (5.9%) patients in the immunochemotherapy group and 15 (14.3%), 83 (79.0%) and 7 (6.7%) patients in the IC-CCRT group achieved complete response (CR), partial response (PR) and stable disease (SD), respectively (p=0.04). No patients experienced disease progression in two groups.

**Figure 1:**
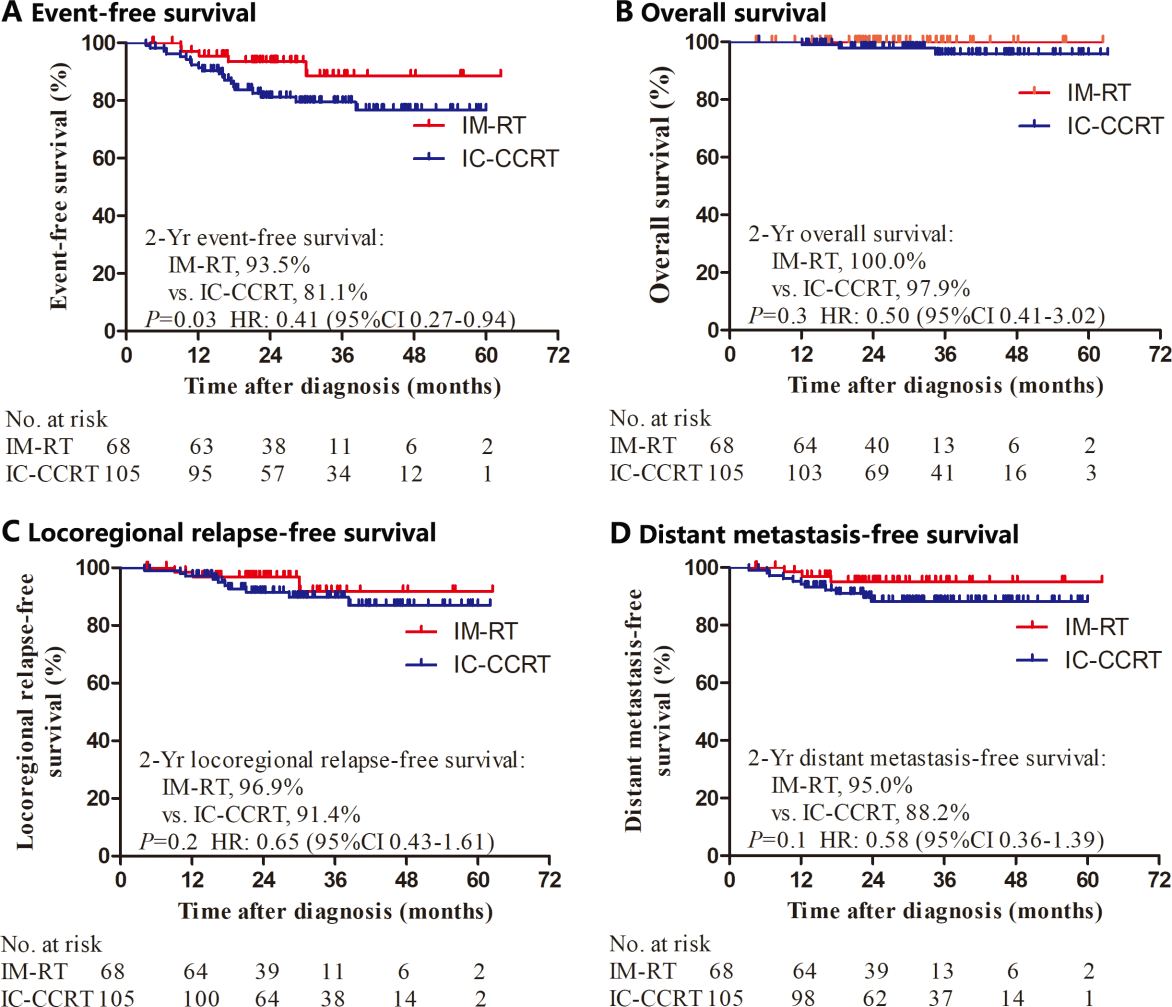
Kaplan–Meier analysis of event-free survival, overall survival, locoregional relapse-free survival and distant metastasis-free survival. IM-RT, immunochemotherapy followed by radiotherapy alone or one cycle of concurrent chemoradiotherapy; IC-CCRT, induction chemotherapy followed by 2 cycles of concurrent chemoradiotherapy.

The median follow-up time was 27.5 months (range, 4.4-63.2). There were 25 patients experienced disease progression or death, including 5 (7.4%, no death) patients in the immunochemotherapy group and 20 (19.0%, 3 death) in the IC-CCRT group. The 2-year EFS was 93.5% in the immunochemotherapy group and 81.1% in the IC-CCRT group (HR, 0.4; 95% CI, 0.17-0.91; p=0.03) (**Figure 1**). The 2-year OS, LRRFS and DMFS rates for the immunochemotherapy group and the IC-CCRT group were 100.0% and 97.9% (p=0.3), 96.9% and 91.4% (p=0.2), 95.0% and 88.2% (p=0.1), respectively (**Figure 1**).

### Adverse events

During induction stage, there were 25 patients (36.7%) who developed acute Grade 3 or 4 adverse events in the immunochemotherapy group and 50 patients (47.6%) in the IC-CCRT group (**Table 2**). The most common acute Grade 3 or 4 adverse events were neutropenia (15 [22.1%] vs. 37 [35.2%]), followed by leukopenia (14 [20.6%] vs. 30 [28.6%]), and nausea (3 [4.4%] vs. 11 [10.5%]) in the immunochemotherapy group and IC-CCRT group (**Table 2**).

**Table 2:** Acute toxicity profile during induction therapy.

Toxicity	Im+IC (N=68) (N, %)	IC (N=105) (N, %)
Major acute event		
G1-2	34 (50.0)	47 (44.8)
G3-4	25 (36.7)	50 (47.6)
Hematological		
Leukopenia		
G1-2	46 (67.6)	67 (63.8)
G3-4	14 (20.6)	30 (28.6)
Neutropenia		
G1-2	28 (41.2)	52 (49.5)
G3-4	15 (22.1)	37 (35.2)
Anemia		
G1-2	36 (52.9)	50 (47.6)
G3-4	1 (1.5)	4 (3.8)
Thrombocytopenia		
G1-2	10(14.7)	30 (28.6)
G3-4	3 (4.4)	8 (7.6)
Non-hematological		
Liver-function		
G1-2	32 (47.1)	46 (43.8)
G3-4	2(2.9)	4 (3.8)
Renal-function		
G1-2	7 (10.7)	9 (8.6)
G3-4	0 (0.0)	0 (0.0)
Nausea		
G1-2	23 (33.8)	55 (52.4)
G3-4	3 (4.4)	11 (10.5)
Vomiting		
G1-2	24 (35.3)	63 (59.4)
G3-4	4 (5.9)	14 (13.3)

Im+IC, immunochemotherapy; IC, induction chemotherapy.

Throughout the entire therapy period, 28 patients (41.1%) in the immunochemotherapy group and 58 (55.2%) in the IC-CCRT group had Grade 3 or 4 adverse events (**Table 3**). Neutropenia was still the most common grade 3 or 4 events (18 patients [26.4%] in the immunochemotherapy group and 42 [40.0%] in the IC-CCRT group). 44.1% of patients experienced grade 1–2 immune-related adverse events in the immunochemotherapy group, and only 5% for grade 3–4 events (**Table 3**). The most common immune-related adverse events were hypothyroidism, pruritus and rashes. The severe irAEs (grade 3-4) were Cutaneous pruritus and Rash.

**Table 3:** Toxicity profile during entire treatment course.

Toxicity	Im+IC (N=68) (N, %)	IC (N=105) (N, %)
Major acute event		
G1-2	36 (52.9)	46 (43.8)
G3-4	28 (41.1)	58 (55.2)
Hematological		
Leukopenia		
G1-2	43 (63.2)	68 (64.7)
G3-4	16 (23.5)	33 (31.4)
Neutropenia		
G1-2	35 (51.5)	51 (48.6)
G3-4	18 (26.4)	42 (40.0)
Anemia		
G1-2	39 (57.3)	52 (49.5)
G3-4	2 (2.9)	5 (4.8)
Thrombocytopenia		
G1-2	13(19.1)	35 (33.3)
G3-4	4 (5.9)	12 (11.4)
Non-hematological		
Mucositis		
G1-2	41 (60.3)	76 (72.4)
G3-4	11(16.2)	24 (22.8)
Vomiting		
G1-2	28 (41.2)	75 (71.4)
G3-4	6 (8.8)	19 (18.1)
Nausea		
G1-2	28 (41.2)	72 (68.6)
G3-4	5 (7.4)	17 (16.2)
Dry mouth		
G1-2	59 (86.8)	94 (89.5)
G3-4	0 (0.0)	3 (2.9)
Dermatitis		
G1-2	60 (88.2)	93 (88.6)
G3-4	3 (4.4)	5 (4.8)
Liver-function		
G1-2	33 (48.5)	49 (46.7)
G3-4	2 (2.9)	6 (5.7)
Renal-function		
G1-2	6 (8.8)	10 (9.5)
G3-4	0 (0.0)	0 (0.0)
Major immune-related event		
G1-2	30 (44.1)	N/A
G3-4	4 (5.9)	N/A
Hypothyroidism		
G1-2	17 (25.0)	N/A
G3-4	0	N/A
Cutaneous pruritus		
G1-2	15 (22.1)	N/A
G3-4	1 (1.5)	N/A
Rash		
G1-2	10 (14.7)	N/A
G3-4	2 (2.9)	N/A
Major late event		
G1-2	59 (86.8)	90 (85.7)
G3-4	5 (7.4)	11 (10.5)
Dry mouth		
G1-2	29 (42.6)	52 (49.5)
G3-4	1 (1.5)	2 (1.9)
Deafness or otitis		
G1-2	10 (14.7)	18 (17.1)
G3-4	1 (1.5)	3 (2.9)
Neck tissue damage		
G1-2	14 (20.6)	27 (25.7)
G3-4	0 (0.0)	0 (0.0)

Im+IC, immunochemotherapy; IC, induction chemotherapy; NA, not applicable.

## Discussion

Concurrent chemoradiotherapy is the landmark in the treatment of locoregionally advanced nasopharyngeal carcinoma (10). Multiple randomized studies have shown that the addition of induction chemotherapy can reduce the tumor size in most patients, eliminate micrometastases, reduce the volume of subsequent radiotherapy, and the patients have good tolerance. Moreover, induction chemotherapy combined with concurrent chemoradiotherapy (IC-CCRT) is currently recommended as the standard treatment regimen for locally advanced nasopharyngeal carcinoma in guidelines (11–16). However, IC-CCRT has a high incidence of short-term toxicity, with more than 70%, and patients have low compliance with the subsequent concurrent chemotherapy (15, 16). Interestingly, a noninferiority, randomized clinical trial reported that after induction chemotherapy, radiotherapy alone was noninferior to chemoradiotherapy in terms of 3-year progression-free survival, with lower short-term toxicity for locoregionally advanced nasopharyngeal carcinoma (7).

To our knowledge, Epstein–Barr virus (EBV) is closely related to the progression of nasopharyngeal carcinoma. Nasopharyngeal carcinoma cells express EBV-related antigens, which could be recognized by T cells. Moreover, tumor tissues are enriched with immune-related cells, such as T cells, B cells, dendritic cells, monocytes and eosinophils (17, 18). These characteristics are theoretically suitable for immunotherapy.

Recently, studies have shown that adding immunotherapy to induction chemotherapy improved tumor response and may be an effective treatment approach for LANPC with good tolerability (19–23). Moreover, additional PD-1 blockade to induction chemotherapy stage and adjuvant stage also leads to significant improvement in tumor regression, cfEBV DNA clearance, superior disease-free survival, and comparable toxicity profiles in high-risk LANPC patients (24). Addition of immune checkpoint inhibitors to chemoradiotherapy improves the survival of patients with locally advanced nasopharyngeal carcinoma, with a 3-year failure-free survival increase of approximately 9% (4, 5). Moreover, the DIAMOND trial has reported that toripalimab combined with induction therapy and radiotherapy alone can reduce toxicity without lowering therapeutic efficacy in LANPC (5). However, the immune checkpoint inhibitors in both of DIAMOND and CONTINUUM trials were applied throughout the entire course, including the induction period, radiotherapy period, and adjuvant period (4, 5). It is still unclear at which stage immunotherapy should be initiated in LANPC.

In view of the toxicities associated with concurrent cisplatin chemotherapy, we compared the efficacy and safety of immunotherapy combined with induction chemotherapy followed by radiotherapy alone or one cycle of concurrent chemoradiotherapy with cisplatin versus induction chemotherapy followed by two cycles of concurrent chemoradiotherapy with cisplatin in patients with locally advanced nasopharyngeal carcinoma in this study. Most patients were at N2 or N3 stage of the lymph nodes or advanced stage of the nasopharyngeal lesion.

Our results showed that the 2-year EFS was 93.5% in the immunochemotherapy group and 81.1% in the IC-CCRT group (HR, 0.4; 95% CI, 0.17-0.91; p=0.03). The 2-year OS, LRRFS and DMFS rates for the immunochemotherapy group and the IC-CCRT group were 100.0% and 97.9% (p=0.3), 96.9% and 91.4% (p=0.2), and 95.0% and 88.2% (p=0.1), respectively. The 100% 2-year OS in the experimental group may be related to the relatively small sample size and the current length of follow-up. Throughout the entire therapy period, 28 patients (41.1%) in the immunochemotherapy group and 58 (55.2%) in the IC-CCRT group had Grade 3 or 4 adverse events. 44.1% of patients experienced grade 1–2 immune-related adverse events in the immunochemotherapy group, and only 5% for grade 3–4 events. The occurrence of grade 3–4 adverse reactions inimmunochemotherapy group was significantly reduced. It’s worth noting that we excluded patients who received adjuvant immunotherapy as we only wanted to explore the role of immunotherapy in the induction stage in LANPC, and the number of patients receiving adjuvant immunotherapy was very small due to COVID-19.

Nevertheless, this study had several limitations. First, this was a retrospective study in single center, and the sample size was small, resulting in potential biases and the limited statistical power. Immunotherapy was not covered by medical insurance. Some patients could not afford the cost of immunotherapy and were concerned about its toxicity, leading to a choice of standard treatment (induction chemotherapy combined with 2 cycles of concurrent chemotherapy). Second, the follow-up duration was short. Third, for a period of time, due to the EBV-DNA testing method, the positive rate of EBV-DNA was low.

After the improvement of testing method, the positive rate became more accurate. The testing methods were inconsistent before and after, so this data is not included in this study.

In summary, compared with induction chemotherapy followed by two cycles of concurrent chemoradiotherapy with cisplatin, immunotherapy combined with induction chemotherapy followed by radiotherapy alone or one cycle of concurrent chemoradiotherapy with cisplatin may improve the 2-year event-free survival of patients with locally advanced nasopharyngeal carcinoma with low adverse events.

However, these results need to be validated in prospective clinical trials.

## Data Availability

The original contributions presented in the study are included in the article/supplementary material. Further inquiries can be directed to the corresponding authors.
